# Online peer support for Young Onset Dementia is a promising resource, but not a panacea: a commentary from the INTERDEM Young Onset Dementia taskforce

**DOI:** 10.3389/frdem.2025.1722518

**Published:** 2025-12-09

**Authors:** Aysegul Humeyra Kafadar, Esther Vera Loseto-Gerritzen, Christian Bakker, Sara Laureen Bartels, David Neal, Jackie Poos, Maud Ritzen, Marjolein de Vugt, Catherine V. Talbot

**Affiliations:** 1 The Institute for Lifecourse Development, University of Greenwich, London, United Kingdom; 2 Institute of Mental Health, School of Medicine, Mental Health and Clinical Neurosciences, University of Nottingham, Nottingham, United Kingdom; 3 Department of Primary and Community Care, and Radboudumc Alzheimer Center, Radboud University Medical Center, Nijmegen, Netherlands; 4 Department of Psychiatry and Neuropsychology and Alzheimer Centrum Limburg, Mental Health and Neuroscience Research Institute, Maastricht University, Maastricht, Netherlands; 5 Department of Clinical Neuroscience, Karolinska Institutet, Stockholm, Sweden; 6 eHealth Living & Learning Lab Amsterdam, Department of Medical Informatics, Amsterdam UMC, Amsterdam, Netherlands; 7 Alzheimer Center Erasmus MC, Department of Neurology, Erasmus MC University Medical Center, Rotterdam, Netherlands; 8 Department of Psychology, Bournemouth University, Bournemouth, United Kingdom

**Keywords:** young onset dementia (YOD), online peer support, online support communities, post-diagnostic support, psychosocial intervention

## 1 Introduction

Approximately 3.9 million people worldwide live with young-onset dementia (YOD), where symptoms occur before the age of 65 (Hendriks et al., 2021). A dementia diagnosis at any age can be challenging for the person and their families, but those under the age of 65 face distinct challenges, including frequent misdiagnosis and the onset of symptoms occurring during a time of heightened financial, work, and family responsibilities (Loi et al., 2023). Therefore, people affected by YOD require age-appropriate care., and Peer support has been recognized as a valuable resource, helping those affected to better cope with symptoms and regain hope, purpose, and belonging (Keyes et al., 2014; Sullivan et al., 2022). However, the availability of specialized YOD services, including in-person peer support groups, varies widely across and within countries, and mainstream dementia services are not always equipped to meet the distinct needs of this group (Mayrhofer et al., 2018). Online peer support offers a promising far-reaching alternative (Gerritzen et al., 2023; Talbot and Coulson, 2023) but a push toward digitalized services risks excluding those without digital skills or resources, potentially exacerbating existing inequalities (Giebel, 2024). Furthermore, increased digitalization could divert funding from essential offline services. As younger, more digitally engaged generation's age, online peer support will become increasingly relevant in dementia care. In this commentary, we, a pan-European taskforce of experts in young-onset dementia, argue that while online peer support can benefit people with YOD, health and social care professionals and support organizations must remain responsive to its challenges to ensure equitable access to support. We call for further large-scale research investigating the effectiveness and implementation of online peer support initiatives to inform best practice in this area.

## 2 Unique benefits of online peer support

Online peer support offers unique benefits compared to in-person groups and may be particularly suitable for people with YOD. Firstly, younger people often have higher levels of digital literacy and are more frequent adopters of digital technologies (Lythreatis et al., 2022), meaning many people with YOD may already possess the skills and devices needed to access online support. Second, online peer support can overcome geographical barriers, which is particularly important given the low prevalence of YOD, making it difficult to connect with peers locally. By connecting people across regions, online peer support can foster a sense of belonging and provide effective coping strategies without the need for travel (Gerritzen et al., 2023, 2022). Third, online peer support provides flexibility, allowing users to choose the format that best suits their needs. Asynchronous platforms, like the YOD sub-forum of the Alzheimer's Society Dementia Support Forum, offer 24/7 access and reach a wide audience facing similar challenges (Talbot and Coulson, 2023). Conversely, videoconferencing (e.g., via Zoom) facilitates real-time interaction within the home environment, creating a sense of togetherness (Gerritzen et al., 2022). This flexibility can allow people affected by YOD to engage with online peer support in their own time and at their own pace, which is crucial for those balancing work, family, and social responsibilities. Lastly, the stigma associated with dementia, especially for younger people who do not fit the stereotypical image, can make support-seeking difficult (Greenwood and Smith, 2016; Giebel, 2021). However, online peer support can offer a degree of anonymity, enabling people to share experiences openly without fear of judgement.

## 3 Potential challenges of online peer support

While online peer support holds promise, health and social care professionals and support organizations must be mindful of several challenges when implementing these initiatives. First, digital inclusion remains a significant issue (Gerritzen et al., 2022). People with YOD living in rural areas and/or facing socioeconomic disadvantages may lack access to suitable devices, reliable internet, or the necessary digital literacy skills to engage with and benefit from online support (Watson et al., 2024). Second, many people with YOD report being unaware of available online support or being unsure of how to find it, highlighting a need for greater awareness of these resources and their benefits (Gerritzen et al., 2023). Third, online peer support raises concerns about safety and security. People with YOD may be vulnerable to scams and misinformation, increasing the caring load of unpaid carers who may need to monitor and support activities (Greenwood and Smith, 2016). Finally, cognitive decline in dementia affects not only memory but also the practical skills required to use technology. As dementia progresses, difficulties with attention, problem-solving, visuospatial skills, and language can hinder engagement with digital platforms, which are typically not designed to accommodate these challenges. For example, commonly used online support spaces on social media often lack features such as simplified navigation, clear visuals, or adaptable communication formats to support people living with dementia (Talbot et al., 2024; Engelsma et al., 2024). Furthermore, rare forms of dementia, for example Frontotemporal dementia and Primary Progressive Aphasia, are relatively more common in younger people (van de Veen et al., 2021) and can come with non-memory-led symptoms such as difficulties with speech and language or vision impairments. Such symptoms could potentially pose additional challenges to using technology and engaging in online communication; however, more research is needed to better understand how different forms of dementia affect engagement with online peer support.

## 4 Discussion

While research investigating online peer support for people with YOD is growing, significant gaps in our understanding remain. Much of the existing evidence is drawn from relatively small-scale qualitative studies [e.g., Gerritzen et al., 2023, 2022; Talbot and Coulson, 2023], which provide valuable information about users' experiences but are limited in their transferability to the wider population of people living with YOD. There is a clear lack of robust quantitative research assessing the effectiveness of online peer support for people with YOD, particularly regarding its long-term impact, the formats which are most effective (e.g., asynchronous vs. synchronous), its effectiveness and adaptability across the disease trajectory, and the contexts in which it is best applied. Furthermore, research on effective recruitment and marketing strategies to raise awareness of online peer support among people with YOD and their families is lacking, which hinders efforts to ensure equitable access to these services. This evidence is essential to inform best practices, guide resource allocation, and support the development of inclusive, evidence-based support models that can be effectively scaled and sustained.

In response to these gaps in evidence and the challenges people with YOD face in accessing online peer support, our pan-European taskforce of experts in dementia proposes the following recommendations:

- Raise awareness of online peer support: Health and social care professionals, dementia advisors, and support organizations should improve signposting to online peer support specifically for YOD.
- Expand online peer support for YOD: Dementia support organizations should develop dedicated online groups specifically designed for people with YOD, to foster a sense of belonging and enable the sharing of similar experiences and effective coping strategies. In support of this work, two of the authors (CT and ELG) are currently conducting a Scoping Review to generate an overview online peer support opportunities for people with dementia, including people with YOD (Loseto-Gerritzen et al., 2025).
- Use trained moderators: Organizers of online support groups should employ trained moderators to facilitate safe, respectful, and supportive interactions. Research with other groups has found moderators can provide additional guidance and reduce online risks (Smedley and Coulson, 2017).
- Improve digital access and literacy: Dementia support organizations and digital poverty initiatives (e.g., Digital Poverty Alliance; Good Things Foundation) provide people with affordable access to digital devices and reliable internet, particularly in rural and socioeconomically disadvantaged areas, and support digital literacy training. By raising awareness of YOD and online peer support in such organizations, they could assist people with YOD in accessing online support services.
- Co-design digital interfaces with people affected by YOD: Technology developers, researchers, and organizations offering online peer support must collaborate with people affected by YOD to co-design platforms that are intuitive and accessible for users with diverse abilities and symptom profiles.
- Expand YOD online peer support research: More research is needed on the long-term impacts and effectiveness of online peer support, to identify support gaps and ensure that emerging technology meets the needs of those affected.
- Maintain a balance between online and offline support: Dementia support services should ensure that online support complements offline services rather than replacing them. Funding for in-person services should be protected to ensure that people who prefer in-person interactions or struggle with digital engagement are not excluded and disadvantaged.

In conclusion, online peer support offers significant potential for people with YOD by overcoming geographical barriers, offering flexibility and anonymity, and fostering community. This is particularly important for people with YOD, who often still balance different roles and responsibilities, such as work and family life, and for whom traditional dementia support groups may not be suitable and may find it particularly challenging to meet peers in their local area. However, the implementation of online peer support services should be approached with care. Challenges relating to digital access and literacy, awareness of available support, online safety, and symptom-related barriers must be addressed. To ensure online peer support is effective and equitable, we recommend using trained moderators, involving people with YOD in co-design, supporting digital device access, offering digital literacy training, and raising awareness of available resources. Additionally, further large-scale research is needed on the design, implementation, and outcomes of online support for people affected by YOD. Importantly, online peer support should complement – not replace – offline support to prevent existing inequalities in dementia care from being exacerbated. By addressing these challenges with thoughtful, inclusive solutions, online peer support can be a powerful tool for improving the lives of people affected by YOD.
